# Treatment of a long-acting anticoagulant rodenticide poisoning cohort with vitamin K1 during the maintenance period

**DOI:** 10.1097/MD.0000000000005461

**Published:** 2016-12-23

**Authors:** Jianhai Long, Xiaobo Peng, Yuan Luo, Yawei Sun, Guodong Lin, Yongan Wang, Zewu Qiu

**Affiliations:** a State Key Laboratory of Toxicology and Medical Countermeasures, Institutes of Pharmacology and Toxicology, Academy of Military Medical Sciences, Beijing, China; b Poisoning Treatment Department, Affiliated Hospital of Military Medical Sciences, Beijing, People's Republic of China.

**Keywords:** LAARs, maintenance treatment, vitamin K1

## Abstract

Currently, there are few guidelines for the use of vitamin K1 in the maintenance treatment of long-acting anticoagulant rodenticide (LAAR) poisonings. We explored factors in the treatment of LAAR poisoning during the maintenance period in order to suggest feasible treatment models.

Data from 24 cases of anticoagulant rodenticide poisoning in our hospital were collected from January 2013 to May 2016. The patients’ sex, age, coagulation function, total time from poisoning to treatment with vitamin K1 (prehospital time), vitamin K1 sustained treatment time (VKSTT), anticoagulant rodenticide category, and specific poison dosage were collected. Multivariate analysis was used to evaluate the correlation between vitamin K1 dosage and other factors during the maintenance period.

Only VKSTT (partial regression coefficient −1.133, 0.59, *P* = 0.035) had an obvious influence on the therapeutic dose of vitamin K1 required during the maintenance period.

After an initial pulse therapy, the bleeding and coagulation functions were stabilized, and the patients were subsequently treated with vitamin K1 during the maintenance period. Over time, the maintenance dose of vitamin K1 (10–120 mg/d, intravenous drip) was gradually decreased and was not related to toxicant concentration.

## Abstract 2

Supplemental Digital Content is available in the text

## 1 Introduction

Long-acting anticoagulant rodenticides (LAARs) have been widely used in agriculture, forestry, and animal husbandry, resulting in an increase in anticoagulant rodenticide poisonings, suicides, and misuse.^[1–3]^ According to the domestic reports of Wang and Jiang,^[4]^ patients with LAAR poisoning accounted for 18% of all poison cases (772/4289) in Jingdezhen City, Jiangxi Province, China from 1996 to 2005. Internationally, similar poisoning cases^[5]^ registered by American Poison Control Centers in 2012 reached 9555 persons. Aside from the fecal–oral route, the most common route of exposure to anticoagulant rodenticide toxicants is absorption through the skin.^[6]^ LAARs can influence the vitamin K cycle by inhibiting vitamin K epoxide reductase (VKOR), resulting in decreased synthesis of hepatic blood coagulation factors II, VII, IX, and X.^[7]^ Clinical examination^[8,9]^ showed that LAARs can significantly prolong prothrombin time (PT) and activated partial thromboplastin time (APTT). The treatment for anticoagulant rodenticide poisoning^[5]^ primarily includes administration of vitamin K1, fresh frozen plasma (FFP), prothrombin complex, and recombinant coagulation factor VIIa. Lubetsky et al^[10]^ proposed that the curative effect of 5 mg vitamin K orally administered is equivalent to 1 mg vitamin K1 intravenously administered. However, there are no oral vitamin K1 preparations available in China. Therefore, an intermittent, long-term, large-dose vitamin K1 intravenous drip^[11]^ is currently used as the main therapeutic schedule for treating LAAR poisoning, with an intramuscular injection as an adjuvant therapy. The highest oral dose of vitamin K1 has been reported to be 800 mg/d.^[12]^ Because LAARs are highly lipid soluble,^[13]^ the measured half-life of rodenticides in vivo tends to be extremely long,^[14,15]^ with an average treatment time of approximately 168 days.^[7]^ There are no commonly accepted methods for detecting anticoagulant rodenticides,^[16]^ including methods using high-performance liquid phase chromatography (HPLC), and no reliable evidence exists describing how to adjust vitamin K dosages.^[17]^ Therefore, there are no accepted standards for the use of vitamin K as a therapeutic agent. In this paper, we analyzed factors that affect the therapeutic dose of vitamin K1 in the treatment of LAAR poisoning in order to provide improved guidance.

## 2 Patients and methods

**Figure 1:**
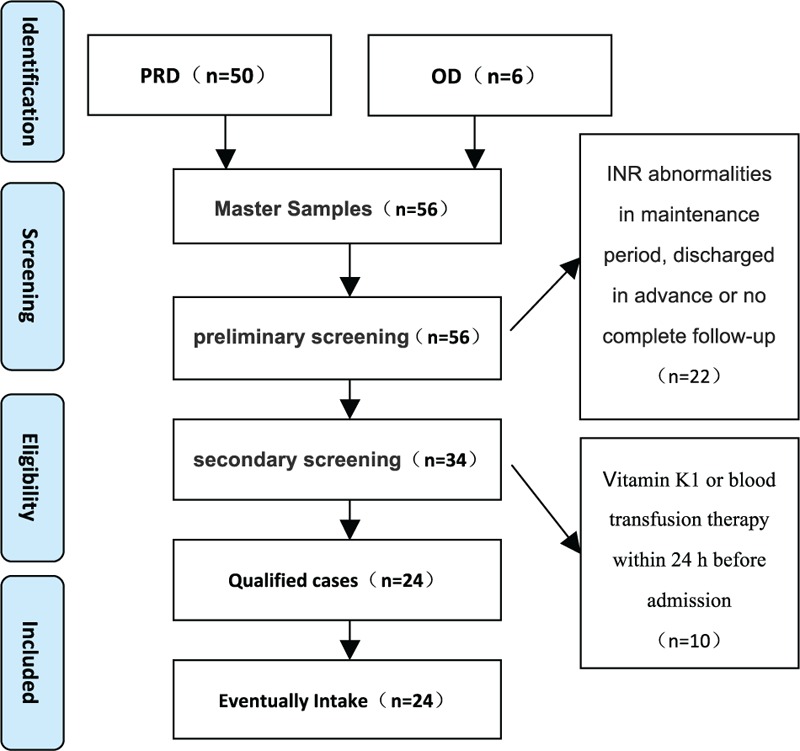
Flowchart of study participants. PRD = poisoning rescue department, OD = other departments.

Patients diagnosed with LAAR poisoning (n = 56) by blood and urine analyses in the emergency department of our hospital from January 2013 to May 2016 were considered for inclusion in this study. Ultimately, 24 cases were included (9 female and 15 male), with an average age of 40.42 ± 19.19 years as shown in Fig. 1. All human participants signed a written informed consent.

Patients who had not received vitamin K1 therapy within 24 h of admission to the hospital, had brodifacoum or bromadiolone detected in their blood or urine, and were treated with a sustained intravenous vitamin K1 drip upon admission to the hospital were included in this study. In addition, included patients had no history of liver disease, had an abnormal international normalized ratio (INR) during the treatment period, and were not currently taking any selective serotonin reuptake inhibitors, nonsteroidal anti-inflammatory drugs (NSAIDs), metronidazole, or cimetidine.^[5]^ Patients who consumed alcohol or had hypoproteinemia during the treatment process were excluded from the study.

Upon admission to the hospital, an initial pulse treatment with vitamin K1 was given to normalize coagulation (INR < 1.5). A maintenance treatment was then initiated, and the type of anticoagulant rodenticide was determined. In addition, the dosage of toxicant, as well as patients’ sex, age, bleeding function, blood coagulation function, INR (reference value 1–1.5), PT activity (PTA, reference value 80%–150%), PT (reference value 8.8–12.8 s), APTT (reference value 24.9–36.8 s), vitamin K1 dosage, prehospital time, and vitamin K1 sustained treatment time (VKSTT) were determined.

For statistical analyses, the vitamin K1 dosage was considered to be a dependent variable. Patient age, coagulation function, brodifacoum exposure, bromadiolone exposure, VKSTT, and prehospital time were considered to be independent variables. Continuous variables were investigated for departure from normality by use of the Shapiro–Wilk *W* test with α = 0.10. For normally distributed outcomes, we conducted a Pearson correlation analysis. For skewed continuous outcomes, we conducted a Spearman correlation analysis. For binary outcomes, we conducted a nonparametric Wilcoxon rank test. Based on the above results, a robust multifactor regression analysis was continuously performed on the selected independent variables (*P* < 0.05). Results were considered to be significant when *P* < 0.05.

## 3 Results

During the study period from January 2013 to May 2016, 56 patients with LAAR poisoning were admitted to the Affiliated Hospital of Military Medical Sciences. Among them, 24 patients were included in this study (Fig. 1) and 32 patients were excluded (10 patients received vitamin K1 24 h before hospital admission; 8 patients had an abnormal INR during the treatment period; and 14 patients were discharged from the hospital ahead of schedule because of economic or family conflicts, resulting in no follow-up examinations).

Table 1 shows baseline characteristics of patients, and Table 2 shows toxicant concentration, hemostasis, coagulation indices, and dosage of vitamin K1 (all of which correspond to the time when blood and urine samples were taken), as well as the patients’ VKSTT (the time from the first day after vitamin K1 was administered until the time of toxicant detection in blood and urine) and prehospital time (the exact time of poisoning or 3 days before the onset of first symptoms^[17]^). This study included 24 patients with an average age of 40.42 years (median, 39 years; range, 12–70 years). Among them, brodifacoum was detected in 7 patients, and bromadiolone was detected in 11 patients; both brodifacoum and bromadiolone were detected in 6 patients. During the maintenance treatment period, the minimum dosage of vitamin K1 was 10 mg/d, the maximum dosage was 120 mg/d, and the median dosage was 60 mg/d.

**Table 1:**
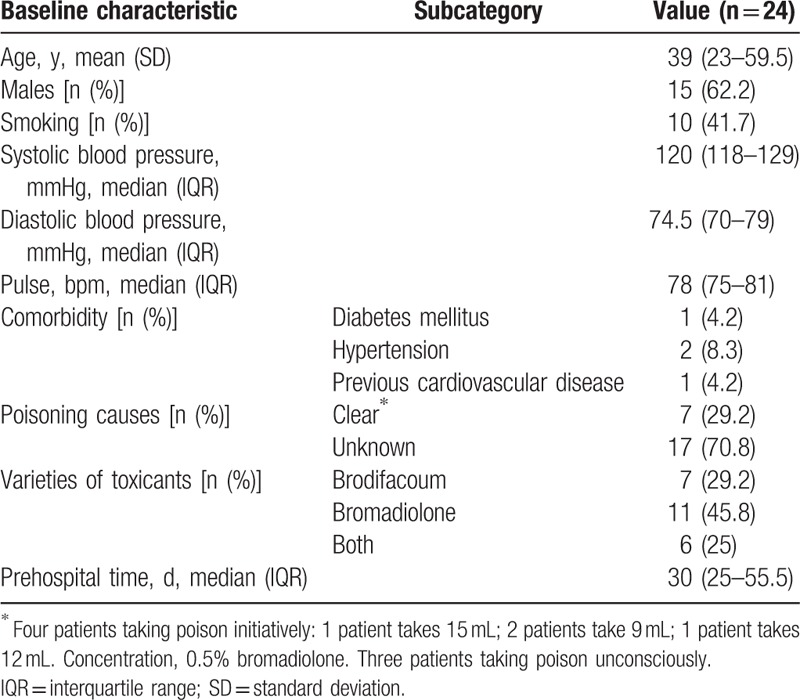
Baseline demographic and clinical characteristics at admission.

**Table 2:**
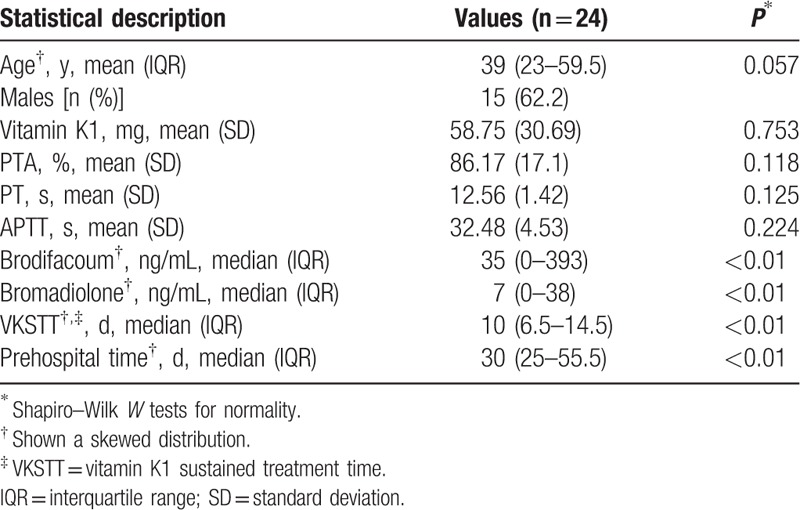
The statistical description of influence factors.

Correlations between multiple factors and vitamin K1 were tested separately (Table 3). Correlations between vitamin K1 and parameters such as PTA, PT, and APTT were analyzed by the Pearson correlation test. Correlations between vitamin K1 and variables such as age, brodifacoum exposure, bromadiolone exposure, VKSTT, and prehospital time were analyzed by the Spearman rank correlation test. Significant correlations were observed among VKSTT, prehospital time, and vitamin K1 (*P* < 0.05).

**Table 3:**
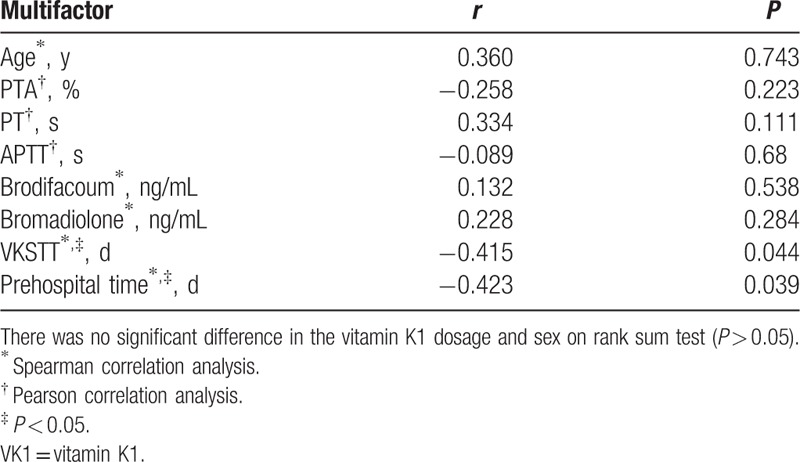
Correlation analysis between multifactor and VK1 dosage.

Because the studentized residuals distribution of the multiple linear regression model did not conform to the residual normality or homogeneity requirements (refer to Supplemental figure, which shows the studentized residuals distribution of the multiple linear regression model), the authors chose a robust regression analysis method to continue the study. As shown in Table 4, only VKSTT (partial regression coefficient −1.133, 0.59, *P* = 0.035) showed statistical significance after the robust regression analysis (Prob > F = 0.0415 < 0.05). The regression equation was as follows: *y*_VK1_ = 81.435 − 1.133*X*_VKSTT_. *P* values for other factors were >0.05 and were not statistically significant.

**Table 4:**
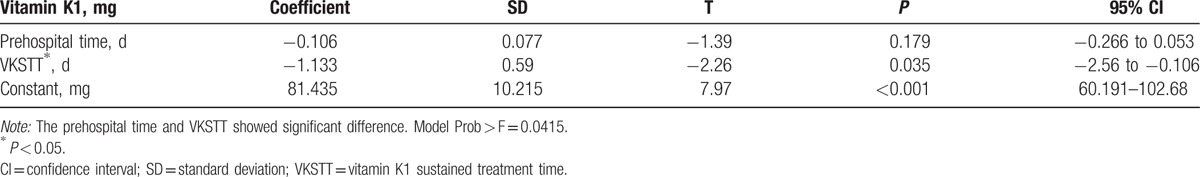
Multifactor robust regression analysis of vitamin K1 dosage.

## 4 Discussion

**Figure 2:**
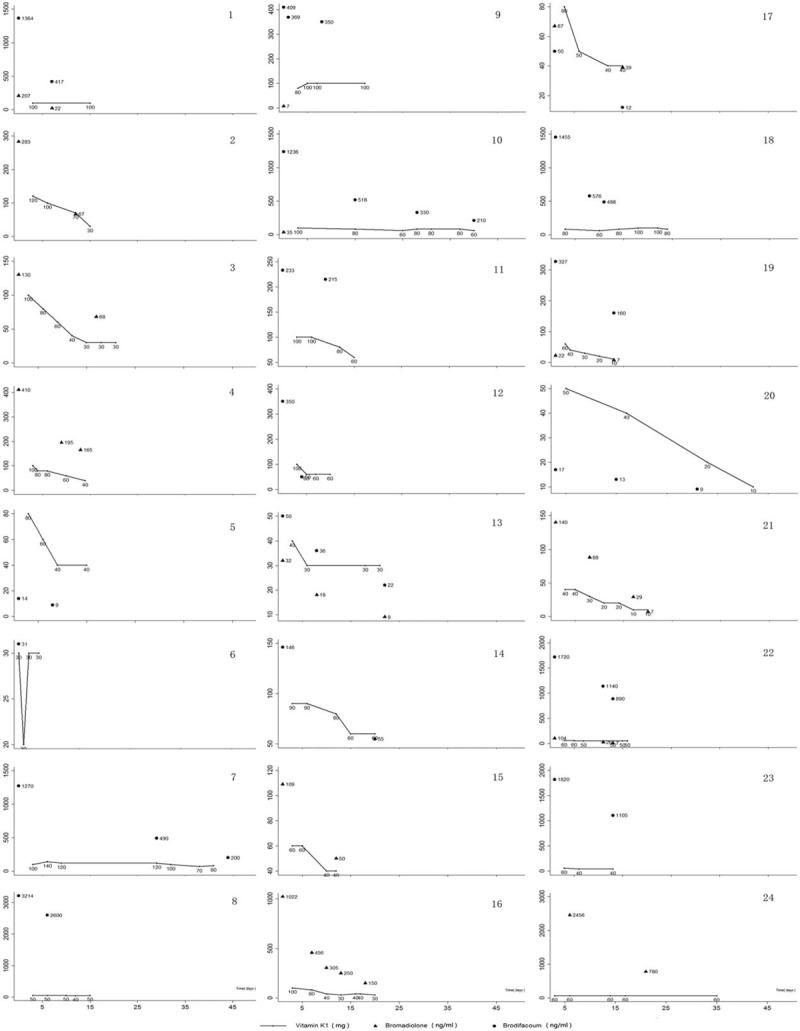
Vitamin K1 dosage for treatment of long-acting anticoagulant rodenticide poisoning during the maintenance period. Data are presented from 24 patients during the maintenance period. The maintenance period is defined as the beginning of the third day of hospitalization, with patients having a normal international normalized ratio. During follow-up, all patients had survived and some completely recovered. However, others continue to receive vitamin K1 treatment.

In this clinical cohort study, the patients were initially treated with large-dose vitamin K1 pulse therapy in order to stabilize the bleeding and coagulation functions. Then, an appropriate dosage of vitamin K1 was adopted as a maintenance therapy as shown in Fig. 2. The concentration of LAAR in patient V was very low; however, a high dose of vitamin K1 (40 mg/d) was still needed. In contrast, the concentration of LAAR in patient V and XXII was high, yet the required dosage of vitamin K1 was similar to that of patient V (40–50 mg/d as static drops), suggesting that there is not a significant dose–effect relationship between the LAAR concentration and vitamin K1 requirements during the maintenance period. These results are consistent with the dosing requirements for vitamin K1 seen in clinical practice. Patient prognoses were good in this cohort, as they all survived. Moreover, the required daily dosage of vitamin K1 (10–120 mg/d, intravenous drip) showed a downward trend that was related to the VKSTT (i.e., vitamin K1 maintenance therapy), but not significantly related to the toxicant concentration.

In patients receiving sustained vitamin K1 treatment, the maintenance dose of vitamin K1 gradually decreased over time as shown by the equation *y*_VK1_ = 81.435 − 1.133*X*_VKSTT_ (effective range: 10–120 mg/d), an effect that was not related to the concentration of LAAR. These results might be attributable to the anticoagulant rodenticide combining with the target of VKOR,^[5,7]^ in which the sustained stimulation of vitamin K1 could increase the expression of VKOR analogs that did not combine with the anticoagulant rodenticides. This would result in a further decrease in vitamin K1 requirements. We did not determine the final concentration of vitamin K1 required by these patients.

Treatments for anticoagulant rodenticide poisonings that have been reported at home and abroad are summarized in Table 5. Gunja et al^[18]^ analyzed the relationship between brodifacoum poisoning and vitamin K1 treatment and reported the INR, poisoning time, brodifacoum concentration, and multidimensional tendency chart of vitamin K1 for 2 cases. Case 1 was treated with a large dose of vitamin K1 (100 mg/d orally administered) sustained over 6 months. When the concentration of brodifacoum reached 5 ng/mL, the vitamin K1 was stopped. Case 2 was treated with a large dose of vitamin K1 (100 mg/d orally administered) sustained over 3 months. When the concentration of brodifacoum reached 4 ng/mL, the vitamin K1 was stopped. Instances of bleeding did not occur in the above patients when the vitamin K treatments were stopped. A large dose of vitamin K1 was continuously administered, so that the real demand for vitamin K1 could not be determined at different time points. Consequently, the results of those case studies provide little information for establishing guidelines, with the primary outcome being a threshold value of 10 ng/mL brodifacoum; once the concentration of brodifacoum reached less than the 10 ng/mL threshold, vitamin K1 treatment could be stopped. In this study, we did not evaluate threshold values because of the low incidence of patients exposed to brodifacoum only. There is no oral vitamin K1 preparation available in China; therefore, we adopted a protocol requiring intramuscular injections of vitamin K1 (10 mg/d) plus follow-up treatments instead of infusion therapy during the maintenance period.

**Table 5:**
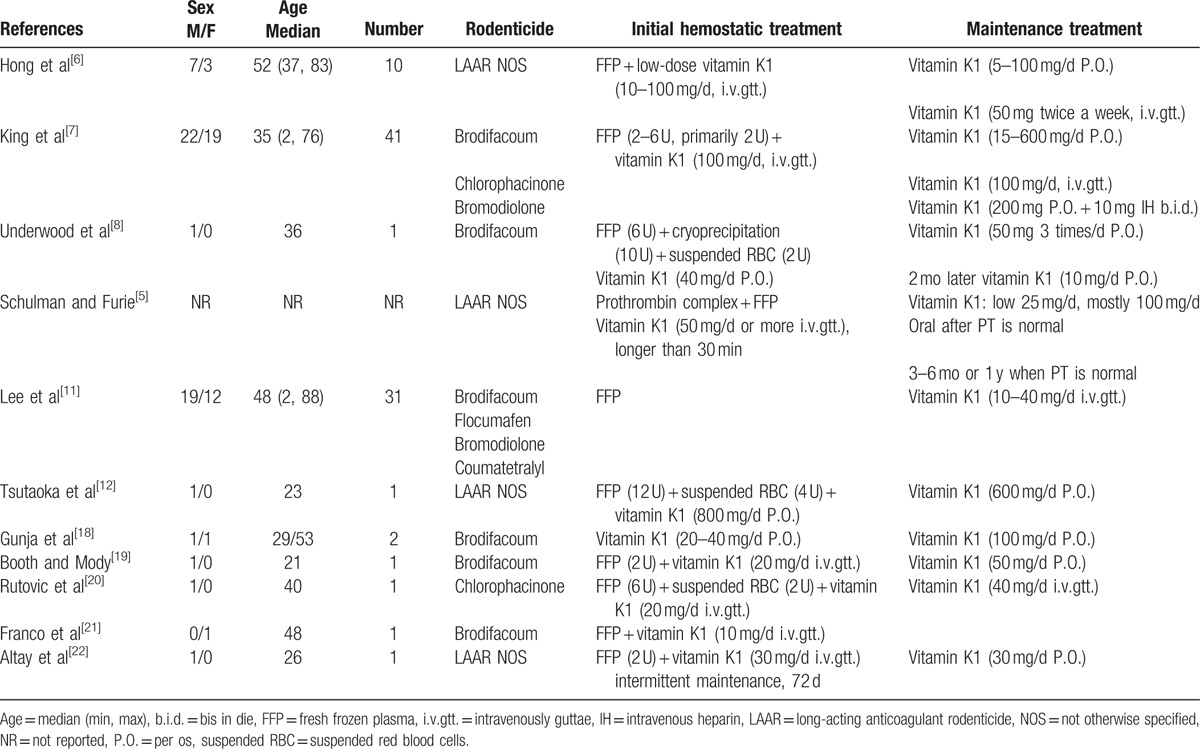
Clinical and treatment data of long-acting anticoagulant rodenticide poisonings.

King et al^[7]^ summarized the treatment of 41 cases of LAAR poisoning. The treatments for each case were similar in the early stages, consisting primarily of FFP (31 cases, 2–6 U, with the majority receiving 2 U) plus vitamin K1 (100 mg/d as an intravenous drip) combined with supportive treatment. During maintenance therapy, vitamin K1 was primarily orally administered at a dosage of 15 to 600 mg/d, with the majority of patients receiving 100 mg/d. Two patients received vitamin K long term by intravenous drip (100 mg/d). A mixed therapy was used in 1 case, in which the patient received 200 mg vitamin K1 orally plus 10 mg subcutaneously twice a day in order to maintain PT at a normal level. The median treatment period was 140 days (average, 168 days; range, 28–730 days).

**Figure 3:**
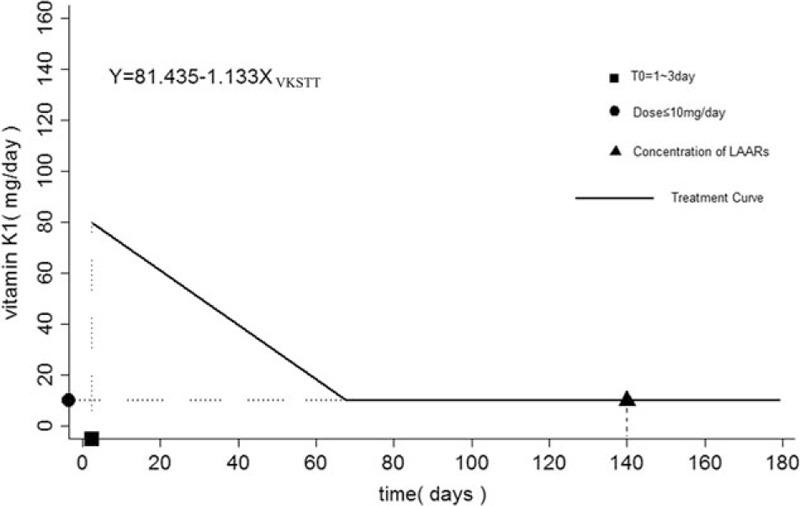
Treatment curve for long-acting anticoagulant rodenticide poisoning. ▪ T 0, 1 to 3 days before maintenance period in which the patient was treated with a prothrombin complex + fresh frozen plasma and vitamin K1 pulse therapy. International normalized ratio or prothrombin time is rapidly restored to normal. ● Vitamin K1 intravenous drip, dosage ≤10 mg/d, long-term low dose maintenance treatment. We did not determine final threshold values. ▴Vitamin K1 treatment was stopped once the concentration of toxicant reached ≤10 ng/mL.^[17]^ VKSTT = vitamin K1 sustained treatment time.

A large number of studies suggest that anticoagulant rodenticide poisonings require a longer maintenance period; however, the suggested maintenance treatments using vitamin K1 present potential risks to patients, and there are no standards of care. For example, intramuscular injections^[23]^ easily cause hematomas, while intravenous administrations^[24,25]^ can result in anaphylactic shock. Further, patient compliance is poor and the poisoning easily relapses, resulting in an increased risk.^[12]^ The present study provides certain guidance for the maintenance treatment of LAAR poisonings and promotes the formation of a standard of care. A treatment curve is shown in Fig. 3.

This study has certain limitations. Genetic differences among patients were not considered in these analyses. Specifically, differences in suppression of the *CYP2C9* and *VKORC1* genes^[26]^ may have resulted in a weakening of LAAR metabolism and an increase in the toxic effects, contributing to an increased risk of bleeding and coagulation. Further, the therapeutic dose of vitamin K1 was limited to 10 to 120 mg/d (intravenous dose q.d.), and the lowest concentration of monotherapy for brodifacoum was 5 ng/mL. There is no further research available on higher or lower therapeutic doses. Finally, only some clinical phenomena were explained, and the therapeutic strategies were investigated by a multifactor regression analysis in this study; therefore, the mechanisms behind these phenomena remain unclear. These results were interpreted based on inferences through published reports, clinical experience, and regression analysis of the data. The results of this study may be attributable to a lack of competitive inhibition between the LAARs and vitamin K1. After successive administration, the distribution of vitamin K1 reached a steady state, and only a small amount of vitamin K1 was required for maintenance treatment. However, successive administrations greater than the minimum dosage resulted in interference (Gunja et al^[18]^ and this study).

## 5 Conclusion

Standardized methods (including HPLC methods) for detecting anticoagulant rodenticides in the blood and urine have not been accepted, and LAAR poisoning occurs mostly in underdeveloped areas.^[27,28]^ Further, the dose of vitamin K1 injections should not exceed 40 mg according to the manufacturer's instructions. The above limitations make the rescue of LAAR poisoning patients a national problem. The results from our robust multifactor regression analysis provide a standardized treatment strategy for anticoagulant rodenticide poisoning. Specifically, successive vitamin K1 treatment was conducted after the bleeding, and coagulation functions were initially stabilized. The vitamin K1 maintenance dosage (10–120 mg/d, intravenous drip q.d.) was gradually decreased over time in a manner that was not related to the poisoning type or concentration of toxicant.

## Supplementary Material

Supplemental Digital Content

## References

[1] Yan S Zhou H Clinical analysis of 48 cases of bromadiolone poisoning. Zhonghua Lao Dong Wei Sheng Zhi Ye Bing Za Zhi 1998;16:377. 22214168

[2] Du X Zhang X Clinical forensic analysis of a population poisoning. Chin J Forensic Med 2003;18:34–5.

[3] Wang X Chen X Diagnosis and treatment of 176 patients with bromadiolone poisoning. Chin Gen Pract 2010;13:1674–5.

[4] Wang Y Jiang J Epidemiological analysis of rodenticides poisoning from 1996 to 2005 in Jingdezhen. Chin J Pest Control 2008;01:25–7.

[5] Schulman S Furie B How I treat poisoning with vitamin K antagonists. Blood 2015;125:438–42. 25377783 10.1182/blood-2014-08-597781

[6] Hong J Yhim HY Bang SM Korean patients with superwarfarin intoxication and their outcome. J Korean Med Sci 2010;25:1754–8. 21165290 10.3346/jkms.2010.25.12.1754 PMC2995229

[7] King N Tran MH Long-acting anticoagulant rodenticide (superwarfarin) poisoning: a review of its historical development, epidemiology, and clinical management. Transfus Med Rev 2015;29:250–8. 26239439 10.1016/j.tmrv.2015.06.002

[8] Underwood EL Sutton J Ellis IK Prolonged coagulopathy after brodifacoum exposure. Am J Health Syst Pharm 2014;71:639–42. 24688037 10.2146/ajhp130537

[9] Nelson AT Hartzell JD More K Ingestion of superwarfarin leading to coagulopathy: a case report and review of the literature. MedGenMed 2006;8:41. PMC1868388 17415322

[10] Lubetsky A Yonath H Olchovsky D Comparison of oral vs intravenous phytonadione (vitamin K1) in patients with excessive anticoagulation: a prospective randomized controlled study. Arch Intern Med 2003;163:2469–73. 14609783 10.1001/archinte.163.20.2469

[11] Lee HJ You MR Moon WR Evaluation of risk factors in patients with vitamin K-dependent coagulopathy presumed to be caused by exposure to brodifacoum. Korean J Intern Med 2014;29:498–508. 25045298 10.3904/kjim.2014.29.4.498 PMC4101597

[12] Tsutaoka BT Miller M Fung SM Superwarfarin and glass ingestion with prolonged coagulopathy requiring high-dose vitamin K1 therapy. Pharmacotherapy 2003;23:1186–9. 14524650 10.1592/phco.23.10.1186.32755

[13] Park J Can we more efficiently save patients with vitamin K-dependent coagulopathy caused by superwarfarin intoxication. Korean J Intern Med 2014;29:430–3. 25045289 10.3904/kjim.2014.29.4.430 PMC4101588

[14] Palmer RB Alakija P de Baca JE Fatal brodifacoum rodenticide poisoning: autopsy and toxicologic findings. J Forensic Sci 1999;44:851–5. 10432620

[15] Hollinger BR Pastoor TP Case management and plasma half-life in a case of brodifacoum poisoning. Arch Intern Med 1993;153:1925–8. 8250654

[16] Fu Z Wang Q Determination of brodifacoum in rat plasma by HPLC. Zhonghua Lao Dong Wei Sheng Zhi Ye Bing Za Zhi 2012;30:135–6. 22808549

[17] Qiu Z Peng X Attention to anticoagulant rodenticides poisoning and Comprehensively improve the level of clinical diagnosis and treatment. Chin J Emerg Med 2014;23:1189–91.

[18] Gunja N Coggins A Bidny S Management of intentional superwarfarin poisoning with long-term vitamin K and brodifacoum levels. Clin Toxicol (Phila) 2011;49:385–90. 21740137 10.3109/15563650.2011.587126

[19] Booth GS Mody PZ Brodifacoum inhalation and its clinical manifestations in a 21-year-old Caucasian man. Lab Med 2016;47:63–6. 26715613 10.1093/labmed/lmv008

[20] Rutovic S Dikanovic M Mirkovic I Intracerebellar hemorrhage caused by superwarfarin poisoning. Neurol Sci 2013;34:2071–2. 23595548 10.1007/s10072-013-1445-2

[21] Franco D Everett G Manoucheri M I smell a rat: a case report and literature review of paradoxical thrombosis and hemorrhage in a patient with brodifacoum toxicity. Blood Coagul Fibrinolysis 2013;24:202–4. 23358203 10.1097/MBC.0b013e328358e959

[22] Altay S Cakmak HA Boz GC Prolonged coagulopathy related to coumarin rodenticide in a young patient: superwarfarin poisoning. Cardiovasc J Afr 2012;23:e9–11. 10.5830/CVJA-2012-051 23108575

[23] Spahr JE Maul JS Rodgers GM Superwarfarin poisoning: a report of two cases and review of the literature. Am J Hematol 2007;82:656–60. 17022046 10.1002/ajh.20784

[24] Mi YN Ping NN Xiao X The severe adverse reaction to vitamin k1 injection is anaphylactoid reaction but not anaphylaxis. PLoS One 2014;9:e90199. 24594861 10.1371/journal.pone.0090199 PMC3942416

[25] Koklu E Taskale T Koklu S Anaphylactic shock due to vitamin K in a newborn and review of literature. J Matern Fetal Neonatal Med 2014;27:1180–1. 24059412 10.3109/14767058.2013.847425

[26] Schalekamp T Brasse BP Roijers JF VKORC1 and CYP2C9 genotypes and phenprocoumon anticoagulation status: interaction between both genotypes affects dose requirement. Clin Pharmacol Ther 2007;81:185–93. 17192772 10.1038/sj.clpt.6100036

[27] Ma J Xu Z One case of mild poisoning by brodifacoum coagulation and its clinical application. Int J Lab Med 2013;34:3272.

[28] Tang L Xu Y Research status of the anticoagulant rodenticide poisoning. J Pediatr Pharm 2012;18:56–9.

