# Supplementary material for: Treatment of a long-acting anticoagulant rodenticide poisoning cohort with vitamin K1 during the maintenance period
Source: Medicine (Baltimore). 2016 Dec 23;95(51):e5461. doi: 10.1097/MD.0000000000005461 (PMC5181810; doi:10.1097/MD.0000000000005461)
Supplement: Supplemental Digital Content [file medi-95-e5461-s001.doc]

Supplemental Figure. Figure that shows the studentized residuals distribution of the multiple linear regression model.


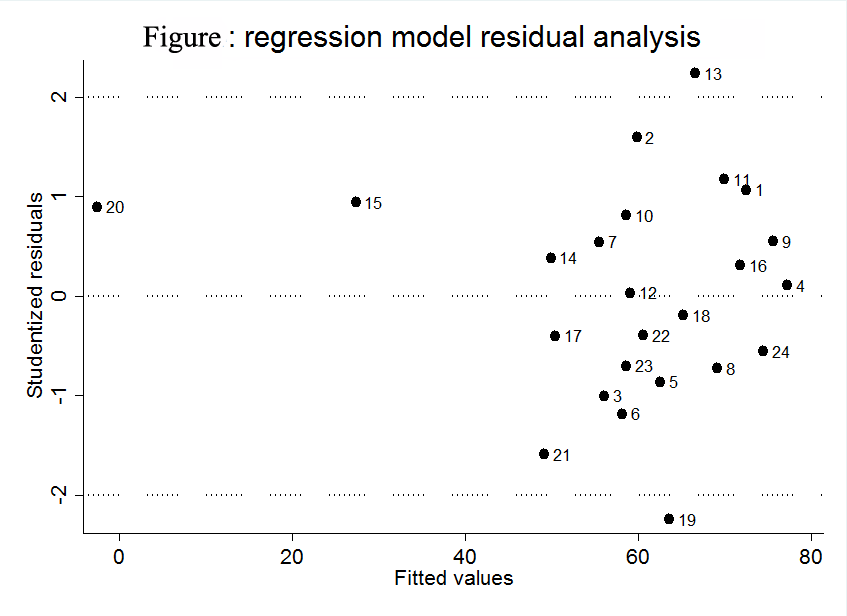


Note：Partial points |r|>2 and studentized residuals distribution did not conform to the residual normality or homogeneity requirements.

The regression analysis was performed with VK1 as the dependent variable, pre-hospital and hospitalization as the independent variable depending on Table 3’s results. Shown in Figure 4, Partial points |r|>2 and studentized residuals distribution did not conform to the residual normality or homogeneity requirements. The robust regression was used for weight allocation according to the different residual size, so that the model was more reliable.
